# Mature-Stage *Eisenia fetida* Proteins Suppress Macrophage Inflammation via NF-κB and MAPK Pathways

**DOI:** 10.3390/ijms27104568

**Published:** 2026-05-19

**Authors:** Hind Althagafi, Hussam A. Althagafi, Fahad Alharthi, Abdullah A. A. Alghamdi, Abdullah M. Almotayri, Ibrahim Jafri, Leena S. Alqahtani, Atif Abdulwahab A. Oyouni, Abdulaziz Albogami, Deyala M. Naguib

**Affiliations:** 1 Department of Biology, College of Science, Princess Nourah bint Abdulrahman University, P.O. Box 84428, Riyadh 11671, Saudi Arabia; 2 Department of Biology, Faculty of Science, Al-Baha University, Al-Baha 65779, Saudi Arabia; 3 Department of Biology, College of Science, Taif University, P.O. Box 11099, Taif 21944, Saudi Arabia; 4 Department of Biotechnology, College of Science, Taif University, P.O. Box 11099, Taif 21944, Saudi Arabia; 5 Department of Biological Sciences, College of Science, University of Jeddah, Jeddah 23445, Saudi Arabia; 6 Department of Biology, Faculty of Science, University of Tabuk, Tabuk 71491, Saudi Arabia; 7 Biodiversity Genomics Unit, Faculty of Science, University of Tabuk, Tabuk 71491, Saudi Arabia; 8 Botany and Microbiology Department, Faculty of Science, Zagazig University, Zagazig 44519, Egypt

**Keywords:** anti-inflammatory, cytokine expression, macrophage, nitric oxide (NO), phagocytosis, reactive oxygen species (ROS)

## Abstract

Earthworm-derived bioactive compounds are emerging as promising pharmaceutical agents; however, the immunomodulatory effects of *Eisenia fetida* proteins at different developmental stages remain unclear. This study evaluated, for the first time, the stage-dependent immunomodulatory activity of *E. fetida* protein extracts in RAW 264.7 macrophages. Soluble proteins isolated from juvenile, mature, and senescent worms were lyophilized and tested for their effects on cell viability, phagocytic activity, nitric oxide (NO), reactive oxygen species (ROS), and inflammatory gene expression. Amino acid profiling and Western blot analysis were additionally performed to investigate biochemical composition and signaling mechanisms. Mature-stage extracts exhibited the highest protein yield, minimal cytotoxicity, enhanced macrophage phagocytosis, and significant suppression of LPS-induced NO, ROS, and proinflammatory cytokines. In contrast, juvenile-stage extracts showed moderate immunomodulatory activity, whereas senescent-stage extracts induced oxidative stress and inflammatory responses. Western blot analysis demonstrated that mature-stage proteins strongly inhibited phosphorylation of NF-κB and MAPK signaling proteins, including p65, IκBα, p38, ERK1/2, and JNK, while senescent-stage extracts maintained elevated pathway activation. Amino acid analysis further revealed enriched immunologically relevant amino acids in mature-stage extracts. These findings demonstrate that developmental stage strongly influences the biological activity of *E. fetida* proteins and highlight mature-stage extracts as promising natural immunomodulatory agents.

## 1. Introduction

Soil fauna, particularly earthworms, are widely recognized as ecosystem engineers due to their crucial roles in organic matter decomposition, nutrient cycling, and the enhancement of soil structure and fertility. Among these, the epigeic earthworm *E. fetida* is of particular ecological and biotechnological interest [1,2,3]. It is not only important for its contribution to vermicomposting and soil health but also for its emerging value as a source of bioactive compounds. Earthworm tissues contain a range of proteins and peptides with reported antimicrobial, antioxidant, and wound-healing properties, indicating potential applications beyond soil ecosystems, including in agriculture, medicine, and environmental biotechnology [4,5,6].

However, little is known about how these bioactivities vary with the physiological age or developmental stage of the earthworm. Aging is known to affect protein synthesis, folding, and degradation, which may alter the functional profile of tissue-derived biomolecules. In addition, little is known about the effect of these compounds as immunomodulators. Studying the immunomodulatory effect of a certain compound is crucial for determining its therapeutic value, mechanism of action, safety profile, and potential application in managing immune-related diseases. This knowledge can ultimately support the development of targeted, effective, and safer treatments [7,8,9]. Thus, understanding how the immunological effects of earthworm protein extracts change over the course of development, from juvenile to mature to senescent stages, may be critical for optimizing their use as bioresources.

In this study, we investigated the immunomodulatory effects of soluble protein extracts obtained from *E. fetida* at distinct developmental stages. Using murine RAW 264.7 macrophages as a model system, we assessed extract cytotoxicity, phagocytic activity, and modulation of inflammatory gene expression following LPS stimulation. This approach aims to elucidate whether the stage-specific protein composition of *E. fetida* influences its biological activity, thereby informing the selection of optimal biomass for future applications in soil health management, plant immunity enhancement, and therapeutic development.

## 2. Results

### 2.1. Eisenia fetida Protein at Different Growth Stages (Juvenile, Mature, and Senescent)

**Figure 1:**
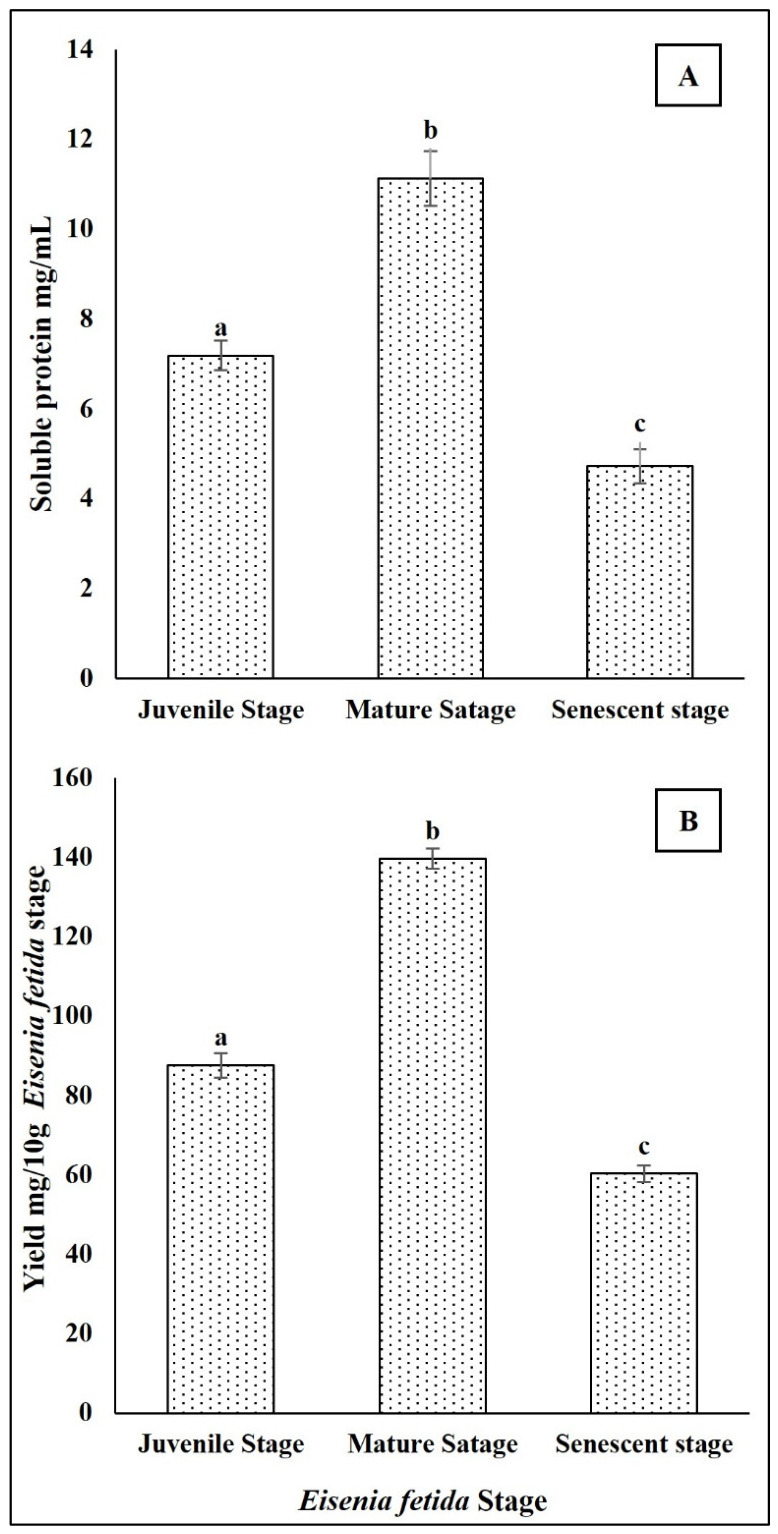
*Eisenia fetida* protein at different growth stages (juvenile, mature, and senescent). (**A**) Soluble protein content and (**B**) protein extract yield. Values are means of three replicates. Error bars represent standard deviation (SD). Values marked with different letters indicate statistically significant differences between groups according to one-way ANOVA followed by Tukey’s HSD post hoc test (*p* < 0.05).

Soluble protein extracts were successfully obtained from *Eisenia fetida* individuals at juvenile, mature, and senescent stages. The protein concentration in the crude extracts, as determined by the Lowry method, showed variation with developmental stage. The mature stage showed the highest protein content with a juvenile stage value of about 4.2 ± 0.3 mg/mL, followed with the juvenile stage with a value of about 6.8 ± 0.4 mg/mL, and the senescent stage showed the lowest protein content with a value of about 3.1 ± 0.2 mg/mL (Figure 1A). The lyophilized powders were fine, white to pale beige in appearance, and stable under storage at −20 °C. The highest yield was obtained from mature worms, consistent with their elevated soluble protein content (Figure 1B).

Amino acid analysis revealed clear developmental stage-dependent differences in Eisenia fetida protein extracts. Overall, mature-stage worms exhibited the richest amino acid profile, whereas senescent-stage extracts showed a marked reduction in most amino acids. Glutamic acid and aspartic acid were the predominant amino acids across all stages.

Most essential amino acids, as well as arginine, histidine, methionine, and cysteine, were more abundant in mature-stage extracts, while senescent worms exhibited generally lower amino acid levels. Proline showed relatively minor variation among stages, with a slight increase in senescent worms. Tryptophan, analyzed separately after alkaline hydrolysis, was detected in juvenile and mature extracts but was not detected in senescent-stage worms. Overall, the mature stage displayed the most balanced and enriched amino acid composition (Table 1).

### 2.2. Effect of Eisenia fetida Protein Extracts on RAW 264.7 Macrophage Cell Viability

**Figure 2:**
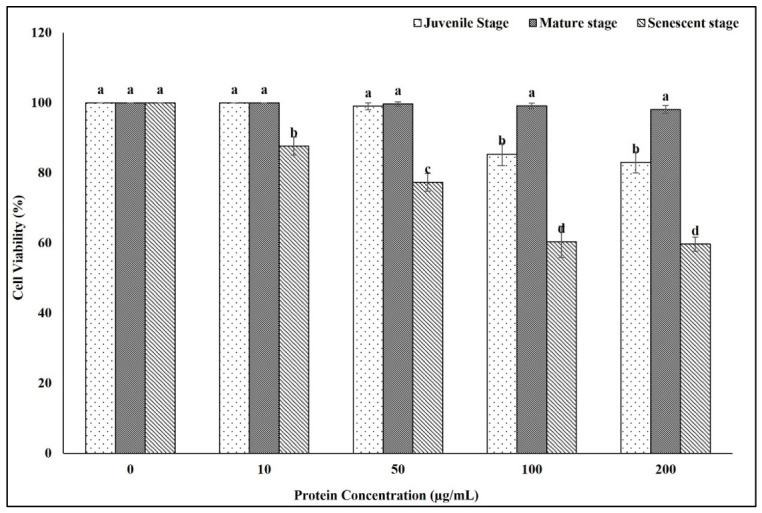
Cytotoxic effects of *Eisenia fetida* protein extracts prepared from juvenile, mature, and senescent worms on RAW 264.7 macrophage. Values are means of three replicates. Error bars represent standard deviation (SD). Values marked with different letters indicate statistically significant differences between groups according to one-way ANOVA followed by Tukey’s HSD post hoc test (*p* < 0.05).

The cytotoxic effects of *Eisenia fetida* protein extracts prepared from juvenile, mature, and senescent worms were evaluated on RAW 264.7 macrophage cells using the MTT assay after 24 h of exposure. The results revealed stage- and dose-dependent differences in cell viability (Figure 2).

Protein extracts from juvenile and mature worms showed minimal cytotoxicity at concentrations of 10–50 µg/mL, maintaining cell viability above 99%. At higher concentrations (100 and 200 µg/mL), the juvenile extract induced a moderate reduction in viability, with values of 85.3% and 83.0%, respectively. In contrast, mature worm extracts exhibited minimal effects even at the highest concentration tested, maintaining cell viability at 98.1%.

In sharp contrast, senescent worm protein extracts demonstrated significant cytotoxicity at all concentrations ≥10 µg/mL. Cell viability decreased markedly to 87.7% at 10 µg/mL, followed by a progressive reduction to 77.3%, 60.3%, and 59.7% at 50, 100, and 200 µg/mL, respectively. These results indicate a distinct toxicity profile associated with the senescent-stage extract, suggesting the presence of bioactive or degradation-related components with higher cytotoxic potential.

### 2.3. Effect of Eisenia fetida Protein Extracts on Macrophage Phagocytic Activity

**Figure 3:**
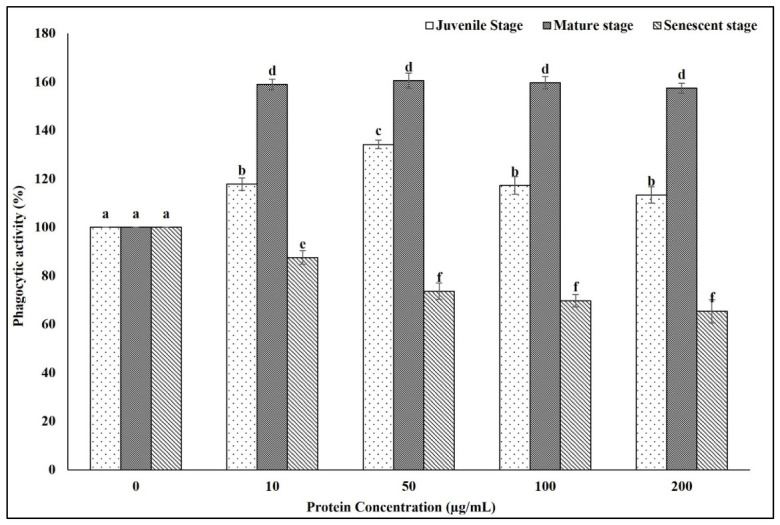
Effect of *Eisenia fetida* protein extracts prepared from juvenile, mature, and senescent worms on RAW 264.7 macrophage phagocytic activity. Values are means of three replicates. Error bars represent standard deviation (SD). Values marked with different letters indicate statistically significant differences between groups according to one-way ANOVA followed by Tukey’s HSD post hoc test (*p* < 0.05).

The effect of *Eisenia fetida* protein extracts from different developmental stages on the phagocytic activity of RAW 264.7 macrophages was assessed using the neutral red uptake assay. As shown in the results (Figure 3), treatment with protein extracts from the mature stage led to a marked increase in phagocytic activity at all tested concentrations, with the highest response observed at 50 µg/mL (160.47%), followed closely by 100 µg/mL (159.67%) and 200 µg/mL (157.33%) compared to the untreated control (100%). Similarly, juvenile-stage extracts exhibited a dose-dependent stimulatory effect, reaching a peak activity of 134.17% at 50 µg/mL, followed by a slight decline at higher concentrations. In contrast, the senescent-stage extracts showed a progressive reduction in phagocytic activity, decreasing from 87.5% at 10 µg/mL to 65.33% at 200 µg/mL. These findings indicate that the immunostimulatory potential of *E. fetida* protein extracts is stage-dependent, with mature-stage proteins exerting the most pronounced enhancement of macrophage phagocytic function.

### 2.4. Effect of E. fetida Protein Extracts from Different Growth Stages on LPS-Induced mRNA Expression of Inflammatory Cytokines and Signaling Molecules in Macrophages

The co-treatment of RAW 264.7 macrophages with LPS and *E. fetida* protein extracts (50 µg/mL) from juvenile, mature, or senescent stages resulted in differential modulation of proinflammatory cytokine gene expression and associated signaling molecules. As expected, LPS treatment alone significantly upregulated the mRNA levels of all tested genes, including *IL-1β* (71-fold), *TNF-α* (16-fold), *IL-6* (32-fold), *NF-κB* (128-fold), *p65* (16-fold), *p38* (64-fold), *ERK1/2* (16-fold), and *JNK* (32-fold) compared to untreated controls.

Co-treatment with juvenile-stage protein substantially downregulated all measured transcripts. Notably, *NF-κB* expression decreased from 128-fold to 10.56-fold, *IL-1β* to 40.22-fold, *TNF-α* to 9.85-fold, and *IL-6* to 8-fold. Similar trends were observed in MAPK signaling components: *p38* and *JNK* levels were reduced to 16-fold and 10.2-fold, respectively.

Treatment with mature-stage protein showed the strongest suppressive effects. All gene expressions were markedly attenuated, with *IL-1β* dropping from 71- to 2-fold, *TNF-α* to 1.41-fold, *IL-6* to 4-fold, and *NF-κB* to 2.38-fold. Corresponding decreases were seen for *p65* (2.83-fold), *p38* (3.36-fold), *ERK1/2* (2-fold), and *JNK* (1.8-fold).

**Figure 4:**
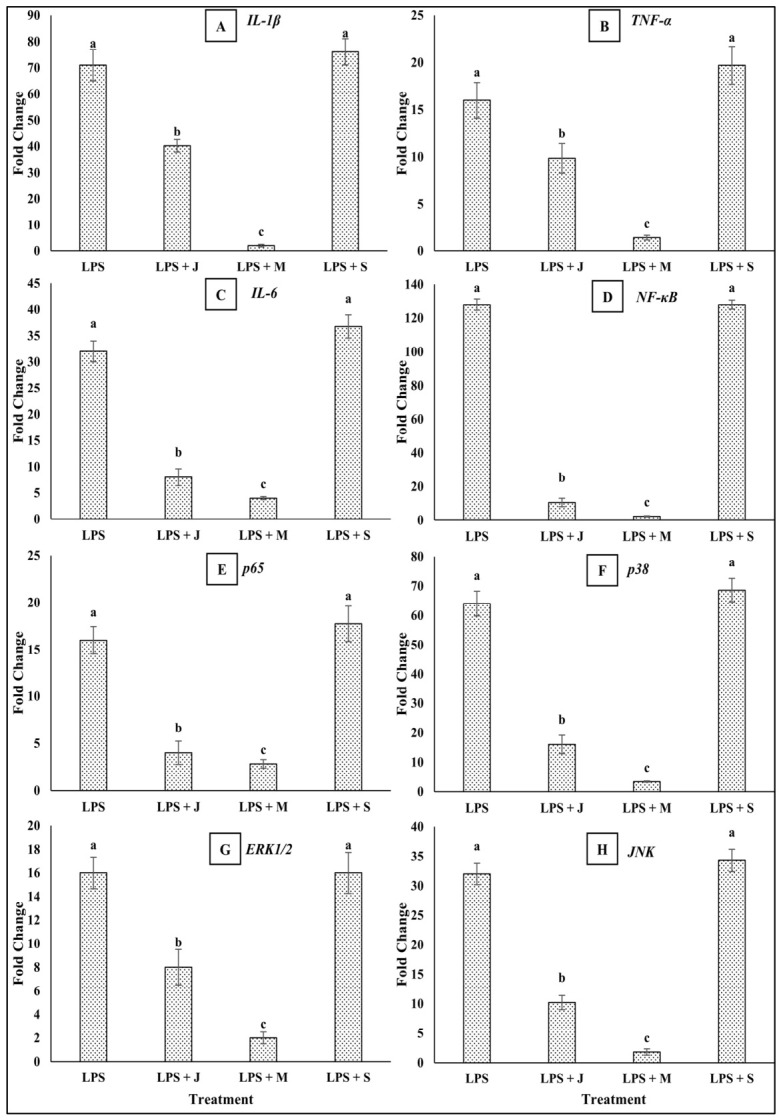
Effect of *Eisenia fetida* protein extracts prepared from juvenile (J), mature (M), and senescent (S) worms on inflammatory cytokines and signaling molecule gene expression (*IL-1β* (**A**), *TNF-α* (**B**), *IL-6* (**C**), *NF-κB* (**D**), *p65* (**E**), *p38* (**F**), *ERK1/2* (**G**), and *JNK* (**H**)) compared with RAW 264.7 macrophages stimulated with LPS. Values are means of three replicates. Error bars represent standard deviation (SD). Values marked with different letters indicate statistically significant differences between groups according to one-way ANOVA followed by Tukey’s HSD post hoc test (*p* < 0.05).

In contrast, the senescent-stage protein either failed to suppress or even potentiated LPS-induced gene expression. In fact, the expression levels of *IL-1β* (76.11-fold), *TNF-α* (19.7-fold), *IL-6* (36.76-fold), *p65* (17.75-fold), *p38* (68.59-fold), and *JNK* (34.3-fold) remained elevated or increased compared to LPS alone. Notably, *NF-κB* and *ERK1/2* levels remained unchanged at 128-fold and 16-fold, respectively. These results (Figure 4) indicate that *E. fetida* proteins from juvenile and mature stages exert anti-inflammatory effects, with the mature-stage extract being the most potent in suppressing LPS-induced proinflammatory gene expression, while senescent-stage proteins may lack such modulatory capacity or potentially exacerbate inflammation.

### 2.5. Western Blot Analysis of NF-κB and MAPK Pathway Activation

**Figure 5:**
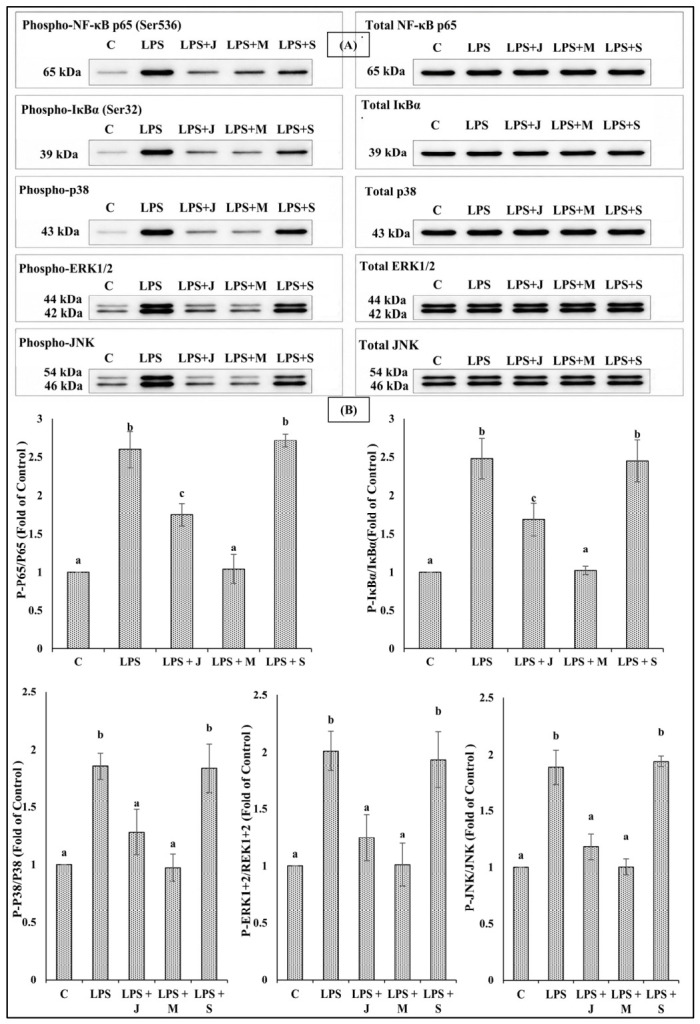
Effects of *Eisenia fetida* protein extracts on NF-κB and MAPK signaling pathways in RAW 264.7 macrophages. (**A**) Representative Western blot images showing the phosphorylation and total protein expression levels of NF-κB p65, IκBα, p38, ERK1/2, and JNK in RAW 264.7 macrophages following treatment with juvenile-stage (LPS + J), mature-stage (LPS + M), and senescent-stage (LPS + S) *E. fetida* protein extracts in the presence of LPS stimulation. Untreated cells (C) served as the negative control, whereas LPS-treated cells served as the inflammatory control. Molecular weights (kDa) of the detected proteins are indicated. (**B**) Densitometric analysis of phosphorylated proteins normalized to their corresponding total protein expression levels and presented as fold change relative to the untreated control group. Data are expressed as mean from three independent experiments, with errors bars representing the SD. Different letters above bars indicate statistically significant differences among groups (*p* < 0.05).

Western blot analysis demonstrated stage-dependent modulation of NF-κB and MAPK signaling pathways in RAW 264.7 macrophages treated with *E. fetida* protein extracts. LPS markedly increased the phosphorylation of JNK, ERK1/2, p38, IκBα, and p65 compared with the untreated control. Juvenile-stage extracts partially reduced pathway activation, whereas mature-stage extracts showed the strongest inhibitory effect, reducing phosphorylation levels to nearly basal control values. In contrast, senescent-stage extracts maintained or further enhanced phosphorylation levels comparable to the LPS-treated group, particularly for p-IκBα and p-p65. These findings indicate that mature-stage proteins exert potent anti-inflammatory effects through suppression of NF-κB and MAPK signaling pathways (Figure 5).

### 2.6. Effect of E. fetida Protein Extracts from Different Growth Stages on Nitric Oxide (NO) Production in Macrophages

**Figure 6:**
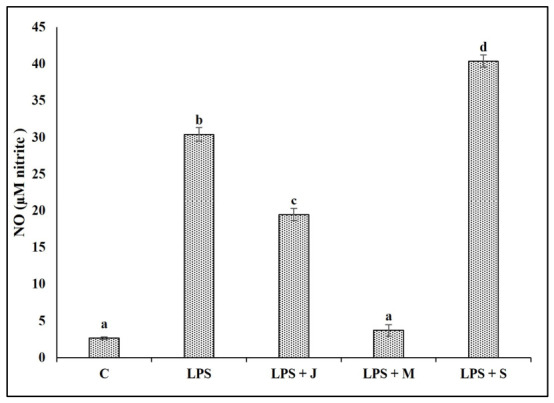
Effect of *Eisenia fetida* protein extracts prepared from juvenile (J), mature (M), and senescent (S) worms on nitric oxide (NO) production in RAW 264.7 macrophages stimulated with LPS. Values are means of three replicates. Error bars represent standard deviation (SD). Values marked with different letters indicate statistically significant differences between groups according to one-way ANOVA followed by Tukey’s HSD post hoc test (*p* < 0.05).

As shown in Figure 6, LPS stimulation significantly increased NO production (30.42 ± 0.94 µM) compared to the untreated control (2.67 ± 0.19 µM), confirming macrophage activation. Pre-treatment with juvenile-stage protein extract (LPS + J) partially suppressed NO levels to 19.46 ± 0.82 µM, indicating moderate anti-inflammatory activity. Mature-stage extract (LPS + M) was most effective, reducing NO production to almost baseline (3.73 ± 0.78 µM), demonstrating strong anti-inflammatory potential. In contrast, senescent-stage extract (LPS + S) not only failed to suppress NO but further increased it to 40.38 ± 0.82 µM.

### 2.7. Effect of E. fetida Protein Extracts from Different Growth Stages on Intracellular Reactive Oxygen Species Production in Macrophages

**Figure 7:**
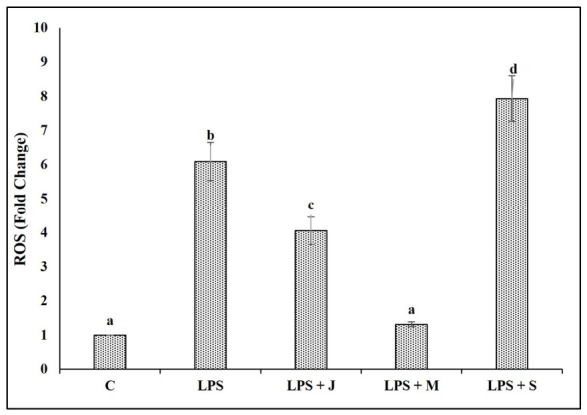
Effect of *Eisenia fetida* protein extracts prepared from juvenile (J), mature (M), and senescent (S) worms on reactive oxygen species (ROS) production in RAW 264.7 macrophages stimulated with LPS (ROS fold change (DCFH-DA fluorescence relative to untreated cells)). Values are means of three replicates. Error bars represent standard deviation (SD). Values marked with different letters indicate statistically significant differences between groups according to one-way ANOVA followed by Tukey’s HSD post hoc test (*p* < 0.05).

As shown in Figure 7, intracellular ROS production in RAW 264.7 macrophages was significantly elevated following LPS stimulation, reaching a 6.1-fold increase relative to untreated cells (SD = 0.56). Pre-treatment with juvenile-stage extract (LPS + J) partially reduced ROS accumulation to a 4.07-fold increase (SD = 0.40), whereas mature-stage extract (LPS + M) produced a pronounced inhibitory effect, lowering ROS levels to near-basal values (1.32-fold; SD = 0.08). In contrast, senescent-stage extract (LPS + S) further heightened oxidative stress, producing the highest ROS levels (7.93-fold; SD = 0.67), exceeding even the LPS-only group. These results demonstrate distinct, stage-dependent effects of *E. fetida* protein extracts on oxidative stress regulation in activated macrophages.

## 3. Discussion

Earthworm tissues (*Eisenia fetida*) contain a diverse array of proteins and peptides, many of which exhibit biological activities such as antimicrobial, antioxidant, anti-inflammatory, and fibrinolytic properties [4,6,10,11]. The soluble protein content of *E. fetida* exhibits developmental stage-dependent variation, reflecting changes in metabolic activity and physiological demands throughout the worm’s growth [12]. The results demonstrate a clear developmental stage-dependent variation in the soluble protein content and its amino acid composition in *Eisenia fetida*, with the mature stage exhibiting the highest values. These findings suggest that the physiological and metabolic state of *E. fetida* varies significantly across its lifecycle, influencing its overall protein content [13]. The peak protein concentration in mature worms can reflect increased biosynthetic activity associated with reproductive maturity, including the synthesis of structural, enzymatic, or reproductive proteins. In contrast, the lower protein levels in juveniles may be due to their still-developing tissue systems, while the decline in senescent individuals may result from decreased protein synthesis or increased protein degradation associated with aging and cellular senescence [14]. Overall, these results underscore the importance of developmental stage in determining the biochemical composition of earthworms and suggest that mature *E. fetida* may serve as the most suitable stage for applications requiring high protein yield, such as biochemical extractions, enzyme characterization, or pharmacological studies.

Earthworm tissues contain a range of proteins and peptides with reported antimicrobial, antioxidant, and wound-healing properties, indicating potential applications beyond soil ecosystems, including in agriculture, medicine, and environmental biotechnology [4,5,6,10,11,15]. Studying the cytotoxicity of specific compounds on macrophages is essential for evaluating their safety and potential immunomodulatory effects, particularly in the context of therapeutic development [16]. The current study investigated the cytotoxic effects of protein extracts from *E. fetida* at different developmental stages on the viability of RAW 264.7 macrophage cells. The MTT assay results revealed stage-dependent differences in cellular responses, with the senescent-stage extract exhibiting markedly higher cytotoxicity compared to juvenile- and mature-stage extracts. Protein extracts from juvenile and mature worms were well tolerated by RAW 264.7 cells, with negligible cytotoxicity observed at concentrations up to 100 µg/mL. Specifically, cell viability remained above 85% for the juvenile extract and above 98% for the mature extract across all tested concentrations, indicating high biocompatibility. These findings suggest that proteins expressed or retained during early and active reproductive stages of *E. fetida* are likely less detrimental to mammalian immune cells, possibly due to their structural integrity, lower content of proteolytic fragments, and reduced accumulation of age-related oxidative modifications. In contrast, protein extracts derived from senescent worms induced a concentration-dependent decrease in macrophage viability, with significant reductions observed even at low concentrations (e.g., ~12% reduction at 10 µg/mL and ~40% reduction at 100 µg/mL). This enhanced cytotoxicity could be attributed to age-related alterations in the protein profile of *E. fetida*, including the accumulation of oxidized or fragmented proteins, elevated levels of inflammatory or degradative peptides, or potential endotoxin-like components released during senescence [17,18]. Previous studies have shown that senescence in invertebrates is accompanied by increased proteolysis and oxidative stress, which may result in bioactive byproducts capable of modulating or impairing mammalian cell viability [19,20,21]. The notable tolerance of macrophages to mature-stage proteins suggests that this stage yields a protein profile with low immunotoxicity, supporting its potential utility in immunological or therapeutic applications. Conversely, the toxicity of senescent-stage extracts underscores the importance of selecting appropriate biological sources when preparing protein-based formulations for biomedical use. Overall, the data highlight a critical influence of organismal age on the biological activity and cytocompatibility of *E. fetida* protein preparations.

Macrophage phagocytic activity is a key indicator of innate immune function and host defense. Studying the effects of specific compounds on macrophage phagocytic activity is crucial for understanding their role in modulating innate immune responses and host defense mechanisms [22,23,24]. The observed enhancement of phagocytic activity in RAW 264.7 macrophages by *E. fetida* protein extracts demonstrates a clear dependence on the developmental stage of the earthworms. The pronounced stimulatory effect exerted by the mature-stage extract suggests that bioactive components involved in immune activation, such as pattern recognition proteins, lectins, or other immunomodulatory peptides, may be maximally expressed or more functionally active during this life stage. The consistent and robust increase in phagocytosis across all tested concentrations in the mature-stage group supports the hypothesis that these worms possess heightened physiological and metabolic activity related to immune defense during reproductive maturity [25,26]. In contrast, the significant and dose-dependent suppression of phagocytic activity by senescent-stage protein extracts may indicate a decline in the production or stability of immune-active compounds with age. Alternatively, aging worms accumulate inhibitory molecules or structural protein fragments [27,28]. These findings are consistent with age-related immunosenescence reported in various invertebrate and vertebrate systems, where the capacity to modulate immune responses diminishes over time [29,30,31]. Taken together, these results highlight the importance of developmental stage in determining the immunomodulatory potential of *E. fetida* proteins. The mature-stage extracts appear particularly promising for further exploration as natural immunostimulants, while the reduced activity of senescent extracts underscores the potential limitations of using aged biological sources in bioactive compound development.

To identify potential anti-inflammatory agents and understand their molecular mechanisms and therapeutic targets for inflammatory and immune-related diseases, it is crucial to study the effect of these agents on LPS-induced mRNA expression in macrophages [32,33]. The present study demonstrates the immunomodulatory effects of *E. fetida* protein extracts from different developmental stages on LPS-induced inflammatory responses in RAW 264.7 macrophages. The gene expression data indicate that protein extracts, particularly from the mature stage, exert potent anti-inflammatory activity, as evidenced by the marked downregulation of proinflammatory cytokines and key signaling molecules. IL-1β, TNF-α, and IL-6 are central proinflammatory cytokines released by activated macrophages during immune responses. These cytokines mediate fever, recruit immune cells to sites of inflammation, and amplify innate and adaptive immune responses. Excessive or prolonged expression of these cytokines is associated with various chronic inflammatory conditions and autoimmune diseases [34,35]. In this study, the mature-stage protein extract significantly suppressed IL-1β (2-fold), TNF-α (1.41-fold), and IL-6 (4-fold) expression compared to LPS alone, suggesting a strong anti-inflammatory capacity. The transcription factor NF-κB (nuclear factor kappa-light-chain-enhancer of activated B cells) plays a pivotal role in regulating the expression of genes involved in immune and inflammatory responses. Its activation leads to the transcription of cytokines, chemokines, and adhesion molecules [36,37,38]. Co-treatment with mature-stage protein drastically reduced NF-κB expression from 128-fold (LPS alone) to 2.38-fold, indicating robust inhibition of this master regulator of inflammation. Similarly, p65, a subunit of NF-κB, was downregulated to 2.83-fold, further supporting suppression of the NF-κB pathway. The MAPK (mitogen-activated protein kinase) pathways—including p38, ERK1/2, and JNK—are also critical for transducing extracellular inflammatory signals to the nucleus. These kinases modulate cytokine production and inflammatory gene expression [39,40,41,42]. The mature-stage extract suppressed p38 (3.36-fold), ERK1/2 (2-fold), and JNK (1.80-fold), reflecting its broad impact on inflammatory signaling cascades. In contrast, the senescent-stage extract either failed to inhibit or even enhanced LPS-induced gene expression. IL-1β, TNF-α, IL-6, p38, and JNK were all elevated compared to LPS treatment alone, suggesting a loss of anti-inflammatory activity or a potential proinflammatory shift in protein composition at the senescent stage. Notably, because the senescent extract caused a partial reduction in cell viability at 50 µg/mL, the increased inflammatory readouts may not reflect direct stimulation alone. Dying or damaged cells can release danger-associated molecular signals, including ATP, HMGB1, and mtDNA, which may activate neighboring cells and amplify ROS, NO, and cytokine production [43]. Therefore, the proinflammatory profile observed for the senescent extract may represent a combined effect of direct bioactivity and secondary signaling from compromised cells.

Importantly, the mature-stage protein extract consistently showed the strongest inhibitory effect across all assessed genes. This stage represents an optimal point in the worm’s lifecycle where bioactive proteins with immunosuppressive properties are most abundant or functionally active [6]. These findings support the hypothesis that proteins derived from mature *E. fetida* possess immunomodulatory potential and could be further explored as natural anti-inflammatory agents. Interestingly, the enhanced phagocytic activity observed in RAW 264.7 macrophages treated with mature-stage *E. fetida* protein was accompanied by a marked downregulation of proinflammatory cytokines and signaling molecules, particularly when co-treated with LPS. This dual activity suggests that the mature protein fraction not only promotes innate immune functionality, evidenced by a ~160% increase in phagocytosis at 50 µg/mL, but also suppresses excessive inflammatory responses through inhibition of IL-1β, TNF-α, IL-6, NF-κB, and MAPK pathway components (p38, ERK1/2, and JNK). Such a profile indicates a shift in macrophage activation towards a more regulated, M2-like phenotype, characterized by efficient phagocytic activity and restrained inflammatory output [44]. This immunological balance is critical for host defense without incurring tissue damage, which underscores the potential of mature *E. fetida* protein as a promising immunomodulatory agent [45,46].

Nitric oxide (NO) is a critical mediator of macrophage-driven immune responses and serves as a key indicator of immunomodulatory activity [47]. In activated macrophages, NO is produced through the inducible nitric oxide synthase (iNOS) pathway in response to proinflammatory stimuli such as LPS. Elevated NO levels reflect classical M1 polarization and are associated with microbial killing, oxidative stress, and amplification of inflammatory cascades. Therefore, compounds that modulate NO production can significantly influence the overall inflammatory state of macrophages [48,49,50,51,52,53,54]. In this study, reduction in LPS-induced NO by juvenile and especially mature *E. fetida* protein extracts indicates inhibition of iNOS expression and upstream transcription factors such as NF-κB and MAPKs, demonstrating strong anti-inflammatory potential. Conversely, the senescent extract’s ability to further elevate NO production suggests proinflammatory or stress-inducing activity. Thus, NO serves as a powerful functional biomarker linking oxidative stress, cytokine regulation, and macrophage activation state [55,56,57].

Reactive oxygen species (ROS) are central regulators of macrophage activation and play a pivotal role in shaping innate immune responses [58,59,60]. Upon stimulation with proinflammatory signals such as LPS, macrophages rapidly increase ROS generation through NADPH oxidase activation and mitochondrial pathways. This oxidative burst functions as a microbicidal mechanism but also serves as a signaling event that amplifies inflammatory pathways, including NF-κB, MAPKs (p38, JNK, ERK), and downstream cytokine production. Therefore, modulation of ROS levels provides direct insight into whether a compound exerts pro- or anti-inflammatory effects [61,62,63,64]. In the present study, the ability of juvenile and especially mature *E. fetida* protein extracts to significantly suppress LPS-induced ROS formation indicates strong antioxidant and immunoregulatory properties. By lowering ROS, these extracts likely prevent the activation of redox-sensitive inflammatory pathways, which explains their simultaneous reduction in NO, inflammatory cytokines (IL-1β, TNF-α, IL-6), and MAPK/NF-κB gene expression [65,66,67,68]. Conversely, the senescent extract’s enhancement of ROS beyond LPS levels suggests a pro-oxidant, proinflammatory profile that aligns with its increased NO production, poor phagocytic performance, and upregulation of inflammatory mediators [69,70].

The Western blot findings strongly support the gene expression results obtained by RT-qPCR, confirming that the immunomodulatory effects of *E. fetida* proteins occur through regulation of upstream inflammatory signaling pathways. LPS stimulation markedly activated NF-κB and MAPK signaling, as demonstrated by increased phosphorylation of p65, IκBα, JNK, ERK1/2, and p38, which is consistent with the elevated expression of proinflammatory cytokines observed at the transcriptional level. Among the tested extracts, the mature-stage proteins showed the strongest inhibitory effects on pathway activation, reducing phosphorylation of both NF-κB and MAPK components to nearly control levels. This suppression closely correlated with the previously observed downregulation of inflammatory cytokine genes, including TNF-α, IL-1β, and IL-6. Since NF-κB and MAPK pathways are major upstream regulators of cytokine transcription, inhibition of these signaling cascades likely explains the reduced inflammatory gene expression [71]. The concurrent reduction in ROS and NO production further supports the ability of mature-stage proteins to suppress oxidative stress-mediated inflammatory signaling. In contrast, senescent-stage extracts failed to inhibit NF-κB and MAPK activation and instead maintained elevated phosphorylation levels comparable to or higher than those induced by LPS alone. This observation is in agreement with the increased expression of proinflammatory cytokines and enhanced oxidative stress detected in macrophages treated with senescent proteins. Persistent activation of p65 and IκBα phosphorylation suggests continuous NF-κB activation, which likely contributes to the cytotoxic and proinflammatory phenotype observed [72,73].

The amino acid profile of *Eisenia fetida* proteins showed clear developmental stage-dependent differences that may explain their distinct immunomodulatory activities. Mature-stage extracts exhibited the richest amino acid composition, particularly in glutamic acid, arginine, essential amino acids, and sulfur-containing amino acids, which are associated with immune regulation, antioxidant defense, and macrophage function. These amino acids may contribute to the suppression of NF-κB and MAPK signaling, reduction in oxidative stress, and regulation of inflammatory cytokine production observed [61,74,75,76]. In contrast, senescent-stage worms showed a marked decline in most amino acids, including methionine, cysteine, and tryptophan, which may contribute to increased oxidative stress, cytotoxicity, and proinflammatory responses. Overall, the findings suggest that the enhanced immunoregulatory activity of mature-stage *E. fetida* proteins is closely linked to their enriched and balanced amino acid composition.

Interestingly, senescent-stage extracts exhibited slightly elevated proline levels compared with juvenile and mature worms. Proline metabolism is closely associated with mitochondrial redox regulation and may contribute to reactive oxygen species generation through proline oxidase-mediated pathways under stress-related conditions [77]. Therefore, the increased proline content observed in senescent worms, combined with the marked reduction in antioxidant-associated amino acids such as methionine, cysteine, and tryptophan, may contribute to the elevated oxidative stress, cytotoxicity, and inflammatory signaling detected in macrophages treated with senescent-stage extracts.

## 4. Materials and Methods

### 4.1. Preparation of Eisenia fetida Protein at Different Growth Stages (Juvenile, Mature, and Senescent)

#### 4.1.1. Protein Extraction

*Eisenia fetida* (IndiaMART InterMESH Ltd.) individuals were categorized into three growth stages based on morphological characteristics: juveniles (no visible clitellum), mature (presence of a developed clitellum), and senescent (aged worms with diminished mobility and faded clitellum). Worms were gently washed with distilled water to remove adhering soil and maintained on moist filter paper for 24 h to allow for gut clearance. Equal fresh weights (10 g) of worms from each stage were homogenized in 50 mM of ice-cold Tris-HCl buffer (pH 7.4) containing a protease inhibitor cocktail (Sigma-Aldrich, Darmstadt, Germany). Homogenization was performed using a tissue grinder on ice. The homogenates were centrifuged at 15,000× *g* for 20 min at 4 °C, and the supernatants were collected as soluble protein extracts. Protein concentrations were determined using the Lowry method [78], and aliquots were stored at −80 °C until use.

The soluble protein fraction was processed to obtain protein powder. Briefly, the crude protein extract was subjected to ammonium sulfate precipitation by adding solid ammonium sulfate to 80% saturation while stirring on ice. The mixture was incubated at 4 °C overnight, followed by centrifugation at 15,000× *g* for 30 min at 4 °C. The resulting protein pellet was resuspended in a minimal volume of distilled water and dialyzed against deionized water for 24 h using a 3.5 kDa MWCO dialysis membrane (Thermo Scientific, Waltham, MA, USA) to remove residual salts. The dialyzed protein solution was then lyophilized using a freeze dryer (Labconco FreeZone, Kansas City, MS, USA) to obtain fine protein powder. The powder was weighed, stored in airtight vials, and kept at −20 °C until further use [79].

#### 4.1.2. Amino Acid Composition Analysis

Amino acid composition of *Eisenia fetida* protein extracts was determined using an automatic amino acid analyzer. Lyophilized protein samples from juvenile, mature, and senescent worms were accurately weighed and hydrolyzed with 6 N HCl in sealed hydrolysis tubes at 110 °C for 24 h under vacuum or nitrogen atmosphere. After hydrolysis, samples were cooled, filtered, and evaporated to dryness under reduced pressure. The dried residues were reconstituted in sodium citrate loading buffer and filtered through a 0.22 µm membrane before analysis. Tryptophan was determined separately after alkaline hydrolysis, because it is degraded during acid hydrolysis [80]. Amino acids were separated using ion-exchange chromatography on an amino acid analyzer, followed by post-column derivatization with ninhydrin reagent. The developed amino acid–ninhydrin complexes were detected spectrophotometrically at 570 nm, while proline and hydroxyproline were detected at 440 nm. Amino acids were identified by comparing their retention times with those of standard amino acid mixtures and quantified using calibration curves prepared from known standards [81]. The results are expressed as g amino acid/100 g protein.

### 4.2. Immunomodulatory Activity Assays

#### 4.2.1. Cell Culture Maintenance and Passaging

RAW 264.7 murine macrophage cells (ATCC^®^ TIB-71™) were cultured in Dulbecco’s Modified Eagle Medium (DMEM) supplemented with 10% (*v*/*v*) fetal bovine serum (FBS). Cells were maintained at 37 °C in a humidified incubator with 5% CO_2_, and the culture medium was replaced every 2–3 days. Subculturing was performed when cells reached approximately 70–80% confluence by gently detaching the cells using a sterile cell scraper, as recommended for this adherent macrophage cell line, and trypsin was not used during passaging. Cells were seeded at appropriate densities following subculturing, and only cells within passage numbers 5–20 were used for all experiments. All cell culture procedures were conducted according to standard protocols based on ATCC guidelines.

#### 4.2.2. Cell Viability Assay (MTT)

Cell viability was assessed using the MTT assay [82]. RAW 264.7 murine macrophage cells (ATCC^®^ TIB-71™) were seeded in 96-well plates at a density of 1 × 10^4^ cells/well and allowed to adhere overnight. Lyophilized *Eisenia fetida* protein powder was reconstituted in sterile phosphate-buffered saline (PBS), filtered through a 0.22 µm syringe filter, and applied to the cells at final concentrations of 10, 50, 100, and 200 µg/mL. Cells were incubated under standard culture conditions as described above for 24 h at 37 °C in a 5% CO_2_ incubator. The 24 h exposure period was selected to allow for sufficient interaction between the extracts and macrophages for detection of cytotoxic effects, consistent with the kinetic requirements of viability assays in RAW 264.7 cells [83]. Subsequently, 10 µL of MTT solution (5 mg/mL in PBS) was added to each well, and the plates were incubated for 4 h. The formazan crystals were solubilized with 100 µL of DMSO, and absorbance was measured at 570 nm using a microplate reader. Cell viability was expressed as a percentage of the untreated control.

#### 4.2.3. Phagocytosis Assay

The phagocytic activity of the macrophages was evaluated using the neutral red uptake method [84]. RAW 264.7 cells were seeded in 96-well plates at 1 × 10^4^ cells/well and maintained under the same culture conditions described above and treated with *E. fetida* protein powder at 10–200 µg/mL for 24 h. This incubation time was chosen to permit measurable modulation of macrophage phagocytic function while minimizing secondary effects associated with prolonged culture, in line with standard RAW 264.7 phagocytosis assays [85,86]. After treatment, 100 µL of 0.075% neutral red solution was added to each well, and the cells were incubated for an additional 1 h. Cells were then washed with PBS to remove excess dye, and the intracellular dye was extracted with a lysis solution containing 50% ethanol and 1% acetic acid. The absorbance was read at 540 nm. The results are expressed as relative phagocytic activity compared to the control.

### 4.3. Molecular Mechanism Studies for Immunomodulatory Effect

#### 4.3.1. mRNA Expression Analysis of Inflammatory Cytokines and Signaling Molecules

RAW 264.7 macrophages were cultured and treated with *Eisenia fetida* protein powder at a final concentration of 50 µg/mL for 6 h. Untreated cells served as the negative control, while cells treated with lipopolysaccharide (LPS, 1 µg/mL) were used as the positive control. The 6 h treatment period was selected to capture early transcriptional responses to treatment before later secondary effects and feedback regulation could occur, which is appropriate for qRT-PCR-based analysis of inflammatory signaling genes [87,88].

Total RNA was isolated from the cells using TRIzol Reagent (Invitrogen, Carlsbad, CA, USA) following the manufacturer’s protocol. Briefly, cells were lysed directly in the culture plate with 1 mL of TRIzol per well (6-well plate) and homogenized by pipetting. Chloroform (0.2 mL per 1 mL TRIzol) was added, and the mixture was vigorously shaken for 15 s and incubated at room temperature for 2–3 min. Samples were centrifuged at 12,000× *g* for 15 min at 4 °C to separate the aqueous phase containing RNA. RNA was precipitated by adding an equal volume of isopropanol, incubated at room temperature for 10 min, and pelleted by centrifugation at 12,000× *g* for 10 min at 4 °C. The RNA pellet was washed twice with 75% ethanol, air-dried briefly, and dissolved in RNase-free water. The quantity and purity of RNA were assessed using a spectrophotometer at 260/280 nm (NanoDrop, Thermo Fisher Scientific, Waltham, MA, USA).

cDNA synthesis was performed using 1 µg of total RNA with a High-Capacity cDNA Reverse Transcription Kit (Applied Biosystems, Foster City, CA, USA) according to the manufacturer’s instructions. The reaction mixture (20 µL) contained RNA template, random primers, dNTPs, reverse transcriptase, and buffer. The reaction conditions were as follows: 25 °C for 10 min, 37 °C for 120 min, and 85 °C for 5 min to inactivate the enzyme. The resulting cDNA was stored at −20 °C until further use.

Quantitative real-time PCR (qRT-PCR) was performed using SYBR Green PCR Master Mix (Applied Biosystems, Foster City, CA, USA) on a StepOnePlus Real-Time PCR System (Thermo Fisher Scientific, Waltham, MA, USA). Each 20 µL reaction contained 10 µL of SYBR Green Master Mix, 0.5 µM of each forward and reverse primer (Supplementary Table S1), 2 µL of cDNA template, and nuclease-free water. The PCR cycling conditions were as follows: initial denaturation at 95 °C for 10 min, followed by 40 cycles of 95 °C for 15 s and 60 °C for 60 s. A melt-curve analysis was performed from 60 °C to 95 °C to confirm the specificity of the amplification.

The expression levels of *IL-1β, TNF-α, IL-6, NF-κB p65*, and MAPK pathway-related genes (*p38, ERK1/2,* and *JNK*) were analyzed. GAPDH was used as an internal reference gene for normalization. Relative mRNA expression was calculated using the 2^−ΔΔCt^ method and expressed as fold change relative to the untreated control. All reactions were performed in triplicate.

#### 4.3.2. Western Blot Analysis of NF-κB and MAPK Pathway Analysis

RAW 264.7 macrophages were cultured and treated with *Eisenia fetida* protein extracts at a final concentration of 50 µg/mL for 6 h. Untreated cells were used as the negative control, whereas lipopolysaccharide-treated cells (LPS, 1 µg/mL) served as the positive inflammatory control. Following treatment, total cellular proteins were extracted using RIPA lysis buffer supplemented with protease and phosphatase inhibitor cocktails. Protein concentrations were determined, and equal amounts of protein (30–40 µg) were separated by SDS-PAGE and electrotransferred onto PVDF membranes.

Membranes were blocked with 5% bovine serum albumin in TBST and subsequently incubated overnight at 4 °C with primary antibodies against phospho-NF-κB p65 (Ser536), total NF-κB p65, phospho-IκBα (Ser32), total IκBα, phospho-p38, total p38, phospho-ERK1/2, total ERK1/2, phospho-JNK, total JNK, and β-actin. After washing, membranes were incubated with horseradish peroxidase (HRP)-conjugated secondary antibodies at room temperature. Immunoreactive bands were visualized using an enhanced chemiluminescence (ECL) detection system and captured using a gel imaging system. Band intensities were quantified using ImageJ software (version 1.54), and normalized to β-actin expression.

#### 4.3.3. Nitric Oxide (NO) Production Assay

Nitric oxide production was quantified using the Griess reagent method [79]. RAW 264.7 macrophages were seeded in 96-well plates at a density of 1 × 10^5^ cells/well and incubated overnight. Cells were pre-treated with earthworm soluble protein extracts from juvenile, mature, or senescent stages (50 µg/mL) for 1 h prior to stimulation with LPS (1 µg/mL). The 1 h pre-treatment period was used to permit early cellular interaction with the extracts before LPS challenge, whereas the subsequent incubation period was selected to allow for measurable NO accumulation in the culture supernatant, in agreement with the time course of inducible NO responses in RAW 264.7 cells [83]. After 24 h of incubation, 50 µL of culture supernatant was mixed with an equal volume of Griess reagent (1% sulfanilamide and 0.1% N-(1-naphthyl) ethylenediamine in 5% phosphoric acid) and incubated for 10 min at room temperature. Absorbance was measured at 540 nm using a microplate reader. Nitrite concentrations were calculated from sodium nitrite standards (0–100 µM). The results were normalized to viable cell number determined by MTT assay.

#### 4.3.4. Intracellular Reactive Oxygen Species (ROS) Assay

Intracellular ROS levels were measured using the DCFH-DA fluorescence method. RAW 264.7 cells were seeded in black 96-well plates (1 × 10^5^ cells/well) and pre-treated with protein extracts (50 µg/mL) for 1 h followed by LPS treatment (1 µg/mL). This short pre-treatment and stimulation schedule was chosen to assess rapid ROS generation, which is an early event in macrophage activation and can be measured within a short exposure window [89]. After 1 h of LPS stimulation, cells were incubated with 10 µM of DCFH-DA in serum-free medium for 30 min at 37 °C in the dark. Cells were washed twice with PBS and fluorescence was measured at 485 nm excitation and 530 nm emission. ROS levels were expressed as fold change relative to untreated control.

### 4.4. Statistical Analysis

All data were analyzed using SPSS software (version 14.0). Results are presented as mean ± standard deviation (SD) based on three independent replicates. Differences among treatment groups were assessed using one-way analysis of variance (ANOVA) followed by Tukey’s Honest Significant Difference (HSD) post hoc test to determine pairwise significance. A *p*-value < 0.05 was considered statistically significant.

## 5. Conclusions

This study demonstrates that the immunomodulatory potential of *E. fetida* protein extracts is highly dependent on worm developmental stage, with mature-stage extracts exhibiting the most promising therapeutic profile. The coordinated action across oxidative, nitrosative, transcriptional, and functional endpoints suggests that mature-stage *E. fetida* proteins exert pleiotropic immunomodulatory effects. In contrast, senescent worms exhibit a loss of regulatory proteins or accumulation of pro-oxidant compounds, resulting in increased oxidative stress, inflammatory cytokine expression, and reduced immune function. Their combined antioxidant, anti-inflammatory, and immunostimulatory properties highlight the potential value of these proteins for applications in immunomodulation, anti-inflammatory therapy, or biologically derived adjuvants. Conversely, the proinflammatory nature of senescent extracts underscores the importance of stage selection in the preparation of biologically active earthworm-derived products.

Future research should aim to characterize the specific protein components responsible for the observed bioactivities using proteomic and bioinformatic approaches. In addition, future studies should include additional positive control treatments to further strengthen assay validation and should employ complementary methods capable of distinguishing between apoptosis and necrosis to better define the mode of cell death associated with cytotoxic responses. Additionally, in vivo studies in animals could further validate the immunological effects and safety of these extracts.

## Figures and Tables

**Table 1:** Amino acid composition in *Eisenia fetida* protein extracts at different stages.

Amino Acid	Juvenile	Mature	Senescent
Aspartic acid	5.047 ± 0.184 ^a^	5.860 ± 0.122 ^b^	3.733 ± 0.252 ^c^
Glutamic acid	7.217 ± 0.225 ^a^	8.497 ± 0.212 ^b^	4.030 ± 0.157 ^c^
Glycine	2.717 ± 0.126 ^a^	3.583 ± 0.189 ^b^	1.450 ± 0.250 ^c^
Alanine	3.270 ± 0.276 ^a^	4.130 ± 0.135 ^b^	7.620 ± 9.856 ^c^
Leucine	5.450 ± 0.250 ^a^	6.590 ± 0.182 ^b^	3.047 ± 0.216 ^c^
Isoleucine	2.833 ± 0.126 ^a^	3.450 ± 0.180 ^b^	1.837 ± 0.182 ^c^
Valine	3.310 ± 0.177 ^a^	3.903 ± 0.150 ^b^	1.967 ± 0.086 ^c^
Lysine	4.543 ± 0.215 ^a^	5.060 ± 0.122 ^b^	2.883 ± 0.275 ^c^
Arginine	5.490 ± 0.300 ^a^	6.823 ± 0.225 ^b^	2.930 ± 0.203 ^c^
Serine	2.387 ± 0.187 ^a^	3.717 ± 0.384 ^b^	1.793 ± 0.199 ^c^
Threonine	2.567 ± 0.161 ^a^	3.080 ± 0.181 ^b^	1.123 ± 0.112 ^c^
Phenylalanine	2.220 ± 0.243 ^a^	3.047 ± 0.112 ^b^	1.483 ± 0.252 ^c^
Tyrosine	1.713 ± 0.198 ^a^	2.560 ± 0.159 ^b^	0.633 ± 0.126 ^c^
Histidine	2.200 ± 0.132 ^a^	3.200 ± 0.180 ^b^	1.563 ± 0.235 ^c^
Methionine	1.013 ± 0.131 ^a^	1.530 ± 0.207 ^b^	0.640 ± 0.101 ^c^
Cysteine	0.530 ± 0.115 ^a^	1.090 ± 0.139 ^b^	0.251 ± 0.073 ^c^
Proline	2.000 ± 0.141 ^a^	2.067 ± 0.161 ^a^	2.960 ± 0.216 ^b^
Tryptophan	0.314 ± 0.142 ^a^	0.863 ± 0.131 ^b^	0.000 ± 0.000 ^c^

Values are means of three replicates ± standard deviation (SD). Values in the same RAW followed with different letters indicate statistically significant differences between groups according to one-way ANOVA followed by Tukey’s HSD post hoc test (*p* < 0.05).

## Data Availability

All data generated in this study is found in this manuscript and its related Supplementary Materials.

## Supplementary Materials

The following supporting information can be downloaded at https://www.mdpi.com/article/10.3390/ijms27104568/s1.
