# Supplementary material for: Mature-Stage Eisenia fetida Proteins Suppress Macrophage Inflammation via NF-κB and MAPK Pathways
Source: Int J Mol Sci. 2026 May 19;27(10):4568. doi: 10.3390/ijms27104568 (PMC13207294; doi:10.3390/ijms27104568)
Supplement: Supplementary file 1 [file ijms-27-04568-s001.zip › ijms-4269711-supplementary.pdf]

Table S1: Genes primer sequencing

Gene	Forward Sequencing primer (5' -> 3')	Reverse Sequencing primer (5' -> 3')
IL-1β	CTCACAGCAGCATCTCGACAAGAG	TCCACGGGCAAGACATAGGTAGC
IL-6	ACTTCCAGCCAGTTGCCTTCTTG	TGGTCTGTTGTGGGTGGTATCCTC
TNF-α	ATGGGCTCCCTCTCATCAGTTCC	GCTCCTCCGCTTGGTGGTTTG
NF-κB	GCTCACAGGCCTACACATACC	GCGATTGCAGAGGTGTTTC
GAPDH	AGCCACATCGCTCAGACA	TGGACTCCACGACGTAC
JNK	TGACTGTCCGTCCTTCTGAA	CCCGGAGCTTCGAAAATTAT
ERK1/2	TCAAGCCTTCCAACCTC	GCAGCCCACAGACCAAA
p38	ACATCGTGTGGCAGTGAAGAAG	CTTTTGGCGTGAATGATGGA
